# Anatomy of the retrohepatic tunnel in a Chinese population and its clinical application in liver surgery

**DOI:** 10.1038/srep44977

**Published:** 2017-03-21

**Authors:** Wang Zheng, Ding Zi-hai, Zhou Jie, Zhong Shi-zhen, Lin Jian-hua, Lin Yi-xiong

**Affiliations:** 1 Anatomical Institute of Minimally Invasive Surgery, Southern Medical University, Guangzhou, China; 2 Department of Genaral Surgery of Guangzhou Integrated Traditional Chinese and Western Medicine Hospital, Guangzhou, China; 3 Department of Hepatobiliary Surgery of Nanfang Hospital, Southern Medical University, Guangzhou, China

## Abstract

Liver hanging maneuver (LHM) is an important technique in liver surgery. However, applied anatomy of the retrohepatic tunnel for the surgical approach in Chinese population needs further study. In this study, to explore the basic anatomy of retrohepatic tunnel and its clinical application in a Chinese population, a total of 32 formalin-fixed cadavers were dissected, related parameters were measured, and their clinical applications were discussed. The length of the retrohepatic tunnel was (60.6 ± 9.9) mm. The width of the retrohepatic tunnel superior opening was (13.8 ± 3.9) mm. The width of the retrohepatic tunnel inferior opening was (15.2 ± 7.4) mm. The hepatic short vessels were distributed along the middle and lower 1/3 of hepatic inferior vena cava (HIVC), with a slight predominance on its left wall. A few hepatic short vessels were distributed along the upper 1/3 of the HIVC. We concluded: the anatomy of the retrohepatic tunnel provides a basis for use of LHM in liver surgery; more hepatic short vessels from hepatic caudate lobe can be preserved via right approach. The retrohepatic tunnel can be used as a good surgical approach in liver surgery; its application also has important significance in laparoscopic minimally invasive liver surgery.

The liver is characteristically rich in blood supply, control of bleeding is often a problem during liver surgery^1^. In China, although advances in liver surgery have kept pace with the rest of the world, this type of surgery has its own particularities. The bleeding control methods used in conventional hepatic resection are obviously insufficient because most of our patients are complicated with cirrhosis of the liver. Furthermore, laparoscopic liver surgery is being rapidly developed in China, the bleeding control methods for this type of surgery are very different from those used in conventional open surgery. In addition, because China has a large population, the number of patients with end-stage liver cirrhosis is much greater than in other countries. The incidence of liver cancer in China is also considerably higher than in the rest of the world. Therefore, more patients in China need liver transplantation than in other countries. Living-donor liver transplantation can potentially benefit more patients than cadaveric liver transplantation due to the shortage of donated livers. All of the above-mentioned procedures require more secure methods to control bleeding during surgery. The use of the anterior approach for liver surgery provides a new solution to this issue. The anterior approach for liver resection was first described by Lai *et al*.^2^, its availability may increase the liver resection rate. However, this approach still has limitations; for instance, because the working space in the deep liver parenchyma becomes smaller during the procedure, it is difficult to control bleeding. The retrohepatic tunnel refers to a potential gap between the hepatic inferior vena cava (HIVC) and the hepatic parenchyma. Belghiti *et al*.^3^ were the first to place a tape through the tunnel to hang the liver up. The name of this technique was translated into Chinese as the “liver hanging maneuver” (hereinafter abbreviated as “LHM”). However, whether or not to extend the use of the LHM remains controversial because the technique was proposed due to concerns about possible uncontrolled bleeding of the injured short hepatic veins (SHV) by blind placement of a hemostat tip into the gap posterior to the liver^4^. We believe that this concern is mainly based on a lack of understanding of vascular anatomy. A consensus regarding the creation of a retrohepatic tunnel does not exist among domestic and international professionals. In particular, detailed descriptions of the anatomy of the retrohepatic tunnel in a Chinese population are lacking. In this article, we studied the feasibility and safety of creating the retrohepatic tunnel using applied anatomy and actual surgical practice.

## Results

### Gross morphology

#### Liver size

The coronal diameter of the liver was (19.54 ± 3.93) (10.49~31.88) cm; the vertical diameter was (13.26 ± 2.44) (8.84~18.73) cm; the anteroposterior diameter was (11.11 ± 2.83) (6.95~19.63) cm.

#### Types of hepatic vein into the IVC

There were 26 cases (81.2%) in which the LHV and MHV shared a common trunk, one case (3.1%) in which the RHV and MHV shared a common trunk, and five cases (15.6%) with individual LHV, MHV, and RHV. Obviously, in the majority of cases, the LHV and MHV possessed a common trunk. These results indicate that it is anatomically possible to create a retrohepatic tunnel in the vena cava fossa.

#### Axial direction of the HIVC

The axis was vertical in 38.7% of cases, shifted to the left of the vertical axis in 38.7% of cases and shifted to the left of the vertical axis in 22.6% of cases.

### Types of vessels draining into the HIVC

#### Inferior-right hepatic vein

IRHVs were present in 28 of 32 liver samples (87.5%). The diameter of the IRHV exceeded 5 mm in eight cases (28.6%). The IRHV diameter was (4.6 ± 3.0) (1.9~14.0) mm. The IRHV was located in the inferior-anterior zone in 25 cases (89.3%), in the middle-anterior zone in one case (3.6%), and in the lower right zone in two cases (7.1%).

#### Posterior right superior edge vein

Posterior right superior edge veins were present in 13 of 32 liver samples (40.6%). Of these samples, the diameter of the posterior right superior edge veins exceeded 5 mm in four cases (30.8%). The diameter of the posterior right superior edge veins was (4.6 ± 2.0) (1.3~8.2) mm. All of the posterior right superior edge veins were located in the superior-anterior zone (100%).

#### Posterior left superior edge vein

Posterior left superior edge veins were present in nine of 32 liver samples (28.1%). The diameter of the posterior left superior edge veins did not exceed 5 mm. The diameter of the posterior left superior edge veins was (3.1 ± 1.3) (1.6~4.5) mm. Of these samples, the posterior left superior edge veins were located in the superior-left zone in five cases (55.6%) and in the posterior superior zone in four cases (44.4%).

#### Right lateral posterior vein

Right lateral posterior veins were present in 7 of 32 liver samples (21.9%). Two large right lateral posterior veins were observed in one case, and only one right lateral posterior vein was present in the remaining six cases. The diameter of the right lateral posterior veins was (5.6 ± 3.4) (2.0~12.6) mm. The diameter of the right lateral posterior veins exceeded 5 mm in four cases (50%). Four of the right lateral posterior veins were located in the middle-anterior zone (50%), and four were located in the inferior-anterior zone (50%).

Of 32 liver specimens, only one case presented large right lateral posterior veins; in this specimen, two veins with diameters of 7.5 mm and 12.6 mm, respectively, were present. In cases of this type, care should be taken to avoid bleeding during separation of the retrohepatic tunnel.

#### Right dorsal vein (only the middle or inferior right, larger SHV are considered in this study)

Right dorsal veins were present in five of 32 liver samples (15.6%). Two right dorsal veins were observed in one case, and only one right dorsal vein was observed in the remaining four cases. The diameter of the right dorsal vein was (2.6 ± 1.0) (1.4~4.0) mm. Three of the right dorsal veins (50%) were located in the middle-right zone, and three (50%) were located in the inferior-right zone.

#### Caudate vein

In 16 of the 32 liver samples (50.0%), only one CV was present; this vein was (3.8 ± 1.2) mm in diameter. In these 16 samples, the CV was located in the middle-left zone in six specimens (37.5%), in the inferior left zone in eight specimens (50.0%), in the middle posterior zone in one specimen (6.2%), and in the inferior posterior zone in one specimen (6.2%). In 13 of the 32 liver samples (40.6%), two CVs were present; these veins were (3.1 ± 1.7) mm in diameter. In these 13 samples, the CVs was located in the middle-left zone in 11 specimens (42.3%), in the inferior left zone in 11 specimens (42.3%), in the superior posterior zone in one specimen (3.8%), and in the middle posterior zone in three specimens (11.5%). In three of the 32 liver samples (9.4%), three CVs were present; these veins were (2.2 ± 0.9) mm in diameter. In these three specimens, one CV was located in the middle-left zone (11.1%), four were located in the inferior left zone (44.4%), one was in the superior posterior zone (11.1%), two were in the middle posterior zone (22.2%) and one was in the inferior posterior zone (11.1%).

In the 32 liver specimens, there were 51 CVs in total. The diameter of the CV was (3.2 ± 1.5) (1.1~8.8) mm. Eighteen (35.3%) of the CVs were located in the middle left zone, 23 (45.1%) were in the inferior left zone, two (3.9%) were in the superior posterior zone, six (11.8%) were in the middle posterior zone and two (3.9%) were in the inferior posterior zone. Two (3.9%) of the CVs were located in the upper 1/3 of the HIVC, 24 (47.1%) were in the middle 1/3 of the HIVC, and 25 (49.0%) were in the lower 1/3 of the HIVC.

In 24 of the liver specimens (75.0%), the CV diameter was greater than 3 mm; two of these 24 cases had two CVs with diameters greater than 3 mm. Of the 51 CVs, 26 (51.0%) had diameters ≧3 mm.

### HIVC

#### Basic measurements

The length of the HIVC was (60.6 ± 9.9) (40.4~81.8) mm. The inner diameter of the HIVC superior opening was (22.9 ± 5.1) (15.0~40.1) mm. The inner diameter of the HIVC inferior opening was (21.8 ± 5.3) (9.3~33.4) mm.

The distance from the RHV opening to the MHV opening or common trunk of the (LHV and MHV) opening was (13.8 ± 3.9) (8.3~22.7) mm. The site between the RHV opening and the MHV opening or the opening of the common trunk of the LHV and MHV is considered the superior opening of the retrohepatic tunnel.

**Figure 1:**
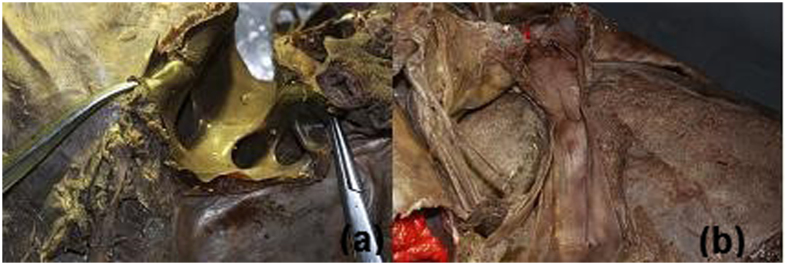
The superior opening of the retrohepatic tunnel (diaphragmatic view) (**a**); the inferior opening of the retrohepatic tunnel (back view) (**b**).

Working space between the IRHV and the CV: The distance from left to right was (15.2 ± 7.4) (3.2~34.3) mm. In 75% of cases, this distance was greater than 1.0 cm. The distance from the top to the bottom of the working space was (10.8 ± 7.3) (0~24.2) mm. The space from left to right between the IRHV and the CV is considered the inferior opening of the retrohepatic tunnel (Fig. 1).

The distance of the upper edge from the LHV or common trunk (LHV and MHV) opening to the RHV opening was (7.7 ± 4.7) (2.0 mm~27.7) mm.

The height of the second hepatic hilum was (19.1 ± 5.7) (10.9~34.9) mm.

The height of the third hepatic hilum was (33.8 ± 9.1) (23.2~55.7) mm.

The distance between the second and the third hepatic hilum was (7.6 ± 5.4) (0~18.0) mm.

The avascular zone was observed in 26 of 32 cases. The avascular zone height was (12.2 ± 4.3) (5.1~24.3) mm. The avascular zone was located in the upper 1/3 of the HIVC in two cases (7.7%), in the middle-upper 1/3 of the HIVC in nine cases (34.6%), in the middle 1/3 of the HIVC in 12 cases (46.2%), in the lower-middle 1/3 of the HIVC in two cases (7.7%), and in the lower 1/3 of the HIVC in one case (3.8%). In 12 of 26 cases (46.2%), the avascular zone was located between the second and third hepatic hilum.

#### Branch type of the vessels into the HIVC

**Figure 2:**
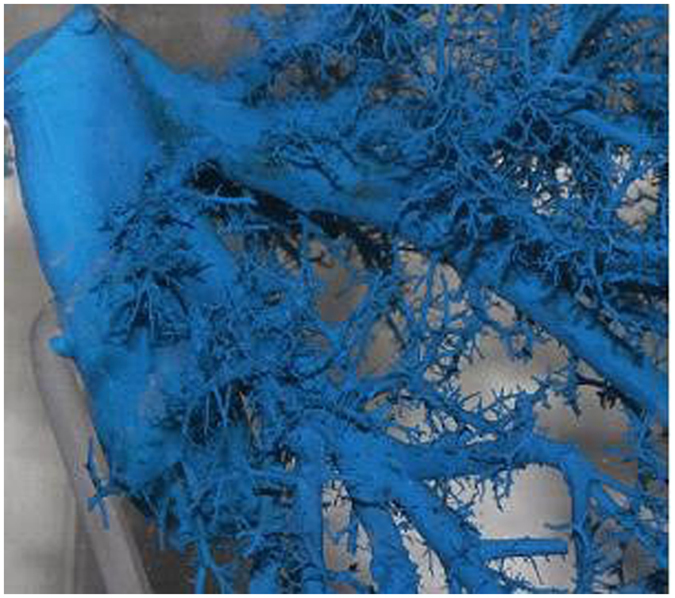
Vascular cast of HIVC.

The origins of the RHV, LHV, MHV, CV, IRHV, right dorsal vein, right lateral posterior vein, right superior posterior vein, and left superior posterior vein included the following four branch types: S: single branch; D: double branches; T: triple branches; Q: quadruple branches (Table 1). The intact morphology of the branches is shown in the cast specimens (Fig. 2).

### Vascular distribution in the retrohepatic tunnel

#### Short hepatic vein

In the 32 liver specimens examined, the number of SHVs in each case was (7.09 ± 4.12) (1~16); the number of SHVs with diameter greater than 1 mm was (5.88 ± 3.17) (1~16).

#### Small vein branches draining into the HIVC (not including the LHV, MHV, RHV, right superior posterior edge vein, or left posterior superior edge vein but including the SHV, CV, IRHV, right lateral posterior vein, and right dorsal vein)

**Figure 3:**
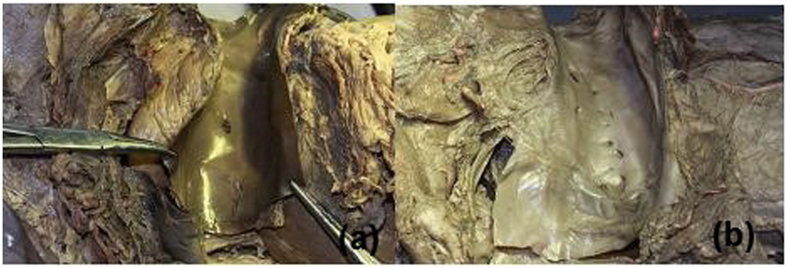
The number of small veins draining into HIVC: 2 (**a**); 20 (**b**).

In the 32 liver specimens examined, the number of the above-defined small veins in each case was (10.00 ± 4.31) (2~20) (Fig. 3); the number of these veins that were greater than 1 mm in diameter was (8.78 ± 3.40) (2~19).

##### Location of the above-defined small veins draining into the IVC

A total of 321 small veins were recorded. Of these small veins, 43 (13.4%) were located in the upper 1/3 of the HIVC, 135 (42.0%) were located in the middle 1/3 of the HIVC, and 143 (44.5%) were located in the lower 1/3 of the HIVC. A total of 281 small veins with diameter greater than 1 mm were recorded in the 32 liver specimens. Of these small veins, 32 (11.4%) were located in the upper 1/3 of the HIVC, 121 (43.1%) were located in the middle 1/3 of the HIVC, and 128 (45.6%) were located in the lower 1/3 of the HIVC.

##### Distribution of the above-defined small veins according to the sub-zones described previously

**Figure 4:**
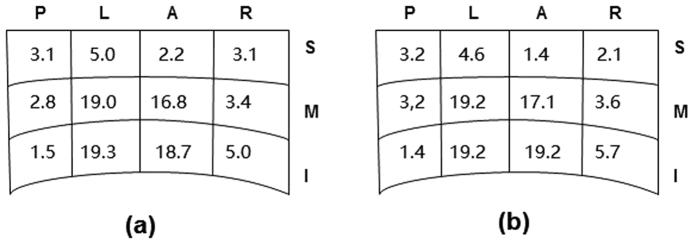
Distribution (%) of all small vein branches draining into the HIVC (**a**); distribution (%) of all small vein branches (diameter ≧1 mm) draining into the HIVC (**b**).

The proportion of small veins located in the superior posterior, left superior, anterior superior, and right superior sub-zones was 3.1%, 5.0%, 2.2%, and 3.1%, respectively. The proportion of small veins located in the middle posterior, left middle, middle anterior, and right middle sub-zones was 2.8%, 19.0%, 16.8%, and 3.4%, respectively. The proportion of small veins located in the lower posterior, left lower, lower anterior, and right lower sub-zones was 1.5%, 19.3%, 18.7%, and 5.0%, respectively (Fig. 4). The proportion of small veins ≧ 1.0 mm in diameter that were located in the superior posterior, left superior, anterior superior, and right superior sub-zones was 3.2%, 4.6%, 1.4%, and 2.1%, respectively. The proportion of small veins ≧ 1.0 mm in diameter located in the middle posterior, left middle, middle anterior, and right middle sub-zones was 3.2%, 19.2%, 17.1%, and 3.6%, respectively. The proportion of small veins ≧ 1.0 mm in diameter located in the lower posterior, lower left, lower anterior, and lower right sub-zones was 1.5%, 19.2%, 19.2%, and 5.7%, respectively (Fig. 4).

## Discussion

Despite advances in our understanding of the anatomy of the liver and surgical procedures, blood loss is still the main complication of liver resection. Massive blood loss is associated with higher postoperative mortality and morbidity^5^. Katz *et al*.^6^ reported that blood loss during liver resection can not only indicate the early postoperative result of liver resection but is also associated with postoperative recurrence of hepatocellular carcinoma and survival. The LHM for liver hepatectomy involves placing a hanging tape through the avascular zone (a plane between 10:00 and 11:00) anterior to the IVC to enable lifting of the liver during liver parenchymal transection and guide the direction of liver resection. This maneuver can help control bleeding during deep parenchymal transection and help avoid injury to the IVC^3^. Liver surgeons worldwide have been familiar with the anterior gap of the IVC in anatomical terms since Belghiti proposed this maneuver in 2001^3^. HOU Dong-sheng *et al*.^7^ believe that the surgical plane between the caudate lobe and the inferior vena cava is located between the inferior vena cava ligament and the caudate lobe capsule. This plane should also be the surgical plane of the anterior space of the HIVC. The left and right margins of the anterior gap of the IVC are the connecting line between the CV and the right edge of the MHV and the connecting line between the right inferior hepatic vein and the left edge of the RHV, respectively. The surgical details of the technique are: (1) the liver is exposed and separated to view the vena cava fossa; (2) the hepatic pedicle is retracted to the upper left to expose the anterior surface of the IVC below the liver; the SHV of the caudate lobe, if there is one, can be divided and ligated; the IRHV can be divided and ligated for right hepatectomy; otherwise, it can be preserved; (3) at the left side of IRHV, a long hemostat can be inserted into the space between the posterior segment of the caudate lobe and the anterior tissue of the IVC; the long hemostat can then be carefully advanced 4–6 cm toward the head side of upper hepatic vein gap; visualization of the hemostat tip in this gap indicates that the retrohepatic tunnel has been formed; (4) using this hemostat, a hanging tape is introduced through the tunnel to surround the liver parenchyma. LHM can be used to solve problems that cannot be solved by the traditional surgical approach, i.e., reduced rates of tumor resection due to tumor invasion of surrounding organs and tumor dissemination due to squeezing of the tumor during the dissection. In addition, the method has the following advantages: (1) it involves the shortest cut-line for liver transection; (2) it provides better exposure of the cut section of the liver, thereby protecting the IVC; (3) safer separation of the MHV is achieved with leftward retraction of the liver. The disadvantage of this method is that it may cause bleeding due to injury to the SHV or the IVC in front of the HIVC. Therefore, this technique is contraindicated if the tumor has invaded the anterior wall of the IVC.

Some researchers attempted to use the LHM for all types of liver resection^8^ regardless of tumor size and whether the tumor was primary or metastatic and found that the use of the LHM did not benefit the overall postoperative results, in particular for total blood loss, blood loss during transection and perioperative blood transfusion rate. Thus, the surgical approach should be selected based on the surgeon’s preference^9^. “Caudate lobe fixation” LHM^10^ for hilar cholangiocarcinoma can also be replaced with the traditional method. In actual practice, the LHM is often used in difficult cases, for instance, cases involving a large tumor or a caudate lobe tumor^11^. The LHM by Glisson’s pedicle approach via the anterior approach can be used for minimally invasive laparotomy hepatic resection^12^. The LHM via the anterior approach can be commonly used for the resection of huge liver carcinoma and of liver carcinoma invading the right diaphragm or the right kidney capsule. The LHM also has the advantage of improving the feasibility of totally laparoscopic hepatic resection^13^ without the hand-assisted approach or open surgery conversion^14^. Robles *et al*.^15^ reported associated liver tourniquet and portal ligation for staged hepatectomy (ALTPS) and achieved good results.

### Criteria for retrohepatic tunnel creation

The upper boundary is the gap (fossa venae cava) between the RHV and the MHV or the common trunk of the MHV and LHV; the lower boundary is the beginning part (from the foot side) of the HIVC (the site where the lowest SHV from the caudate lobe drains into the IVC). The connecting lines of both of the ends between the upper and lower boundary lines are the left and right boundary lines. Thereby, a tunnel with a narrow upper opening and a wide lower opening, also known as a “retrohepatic tunnel”, is formed between the liver parenchyma and the HIVC. According to empirical clinical practice, the width of the upper boundary is 0.6 cm, and the width of the lower boundary is 1.0 cm; the space between the upper and the lower boundaries is the same as the space of the retrohepatic tunnel where the long hemostat tip was inserted. According to our measurement results, the distance from IRHV to the largest CV was (15.2 ± 7.4) mm. In 75% of cases, this distance was greater than 1.0 cm. The hemostat tip needs to move in 1.0 cm of range in the inferior opening. In most cases (75%), this requirement can be met without ligation of the CV and IRHV. Otherwise, the CV or IRHV can be ligated to meet this requirement according to the range of the surgical resection; the space between RHV and MHV or common trunk (LHV and MHV) of every case was over 6 mm. The hemostat tip needs to move in 0.6 cm of range in the superior opening. All up to the requirements. The results above indicate that most of the retrohepatic tunnels are sufficient for a forceps to pass through. It has been reported that there is a 1-cm-wide avascular zone between the IRHV and the largest caudate lobe vein in front of the HIVC; the RHV, the IRHV, and the MHV drain into the IVC on its right wall, whereas the CVs drain into the IVC on its left wall. This current study demonstrated that such avascular zone did not exist because of the presence of relatively small vessels; however, the vein locations were similar to those described previously.

The literature^16^ reported observation of the IRHV in 84% of cadaveric livers, similar to the 87.5% observed in this study. The IRHV should be preserved to protect the blood supply and function of the residual liver if the IRHV does not supply the resected liver segment. The large and thick IRHV is even the blood flow compensation for Budd-Chiari syndrome. Visualization of the IRHV, was approximately 28.33% in Doppler ultrasound and approximately 30% in MSCT. Because the IRHV is the relatively large vessel in the retrohepatic tunnel, it appears that current imaging methods is easy to ignore the existing small vessels for preoperative assessment. In clinical practice, the vessels in the retrohepatic tunnel are mainly detected and managed during surgical exploration. Therefore, it is very important to have anatomical consensus for the vessels in the retrohepatic tunnel.

During LHM, large veins should be protected or managed individually. However, the management of the CVs remains controversial. Some researchers believed that the purpose of studying the small vessels, such as the left caudate SHV and the right caudate lobe SHV, which are not the major vessels draining the caudate lobe, is to improve bleeding control during the blind passage of the hemostat tip. Sato^17^ demonstrated that a large amount of smooth muscle is present in the outer membrane of the IVC and that bleeding of the small hepatic veins can be controlled by stimulating this smooth muscle. The majority of researchers believe that 1 mm is the minimum diameter for veins to be considered clinically significant^18^. However, the studies of Mehran R *et al*.^18^ showed that the SHV partially or completely drain liver segments I, VI, VII, and IX. The pathophysiological importance of the SHV is even more meaningful in the setting of poor drainage of the major hepatic veins, such as in Budd-Chiari syndrome or in cases of giant tumors of the liver. Liver scanning with technetium radioisotope showed that the SHV drain blood from the functional segment of the liver into the IVC in patients with Budd-Chiari syndrome. Therefore, we speculate that the presence of SHV is significant and that 1-mm or larger branches should be preserved as much as possible if it not necessary to ligate them. From the viewpoint of embryogenesis, the caudate lobe is a reserve/compensatory liver lobe with its own bile duct system. This highlights the significance of the presence of the short hepatic vessels of the caudate lobe of the liver.

Hirai I *et al*.^19^ described three approaches to pass the hemostat tip through the retrohepatic tunnel: the right approach, the middle approach and the left approach. Two marker points were used to identify each of the approaches. A common marker point is located in the center of the vena cava fossa; the other marker points in the lateral side of the liver may be at various locations: the right caudate process or S6 process is the marker to identify the right approach, the deepest point of the gallbladder bed is used to identify the left approach, and the middle point between the first two points identifies the middle approach. These authors suggested preserving not only the atypical CVs but also the typical CVs so as to maintain caudate lobe function after surgery. In cases in which the IRHV is absent, the right approach to creation of a retrohepatic tunnel is preferred because it allows more of the CVs to be preserved. LHM can be used for enlarged left liver graft preparation during living donor liver transplantation because injury of the left caudate portal branch in the main cutting section rarely occurs in this setting. Belghiti’s LHM seems very practical for right or left hepatectomy in the treatment of hepatocellular carcinoma, liver metastases, benign tumors, and traumatic liver injury as well as enlarged left lobe grafts harvested during living-donor liver transplantation. In short, the right approach seems to be the best approach for passage of the hemostat tip through the tunnel because it is rare to injure the CV by this approach. To preserve the CVs and the IRHV, it is recommended that the hanging tape be bundled up and tightened up toward the right of the IRHV or that the hemostat tip direction be changed from toward the left to toward the right when the tip is in the front of the IVC. The LHM without rotation of the liver is useful and convenient for both right and left hepatectomy in the setting of giant liver tumor or cirrhosis of the liver.

**Figure 5:**
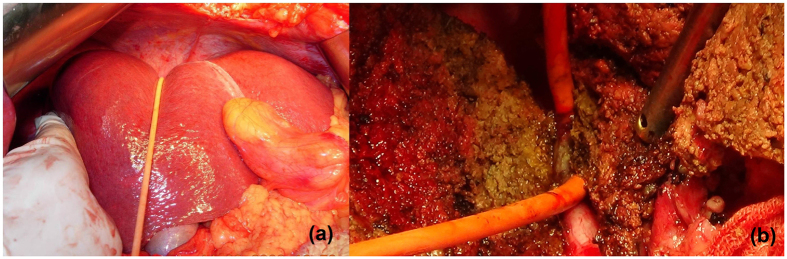
The hanging tape was placed through the retrohepatic tunnel to pull the liver for bleeding control (**a**) and guide the liver parenchyma transection (**b**).

We recommend that the anterior approach be used for complex and difficult liver resections, for instance, liver carcinoma >5 cm; in addition, the LHM can make it easier to control bleeding and provide better exposure of the operative field. Some researchers argue that using the approach can meet the principle of tumor resection, but it is difficult to perform. However, we believe that using this approach does not increase the difficulty of the operation when undertaken by a skilled surgeon with a clear understanding of liver anatomy (Fig. 5). At the time of creation of the retrohepatic tunnel, the third hepatic hilum is delt with from the bottom up and the hanging tape is placed around; this is in accordance with the laparoscopic vision and operation habits. During laparoscopic right hemi-hepatectomy, first the retrohepatic tunnel can be created and a hanging tape can be placed to lift the liver; the combined application of the anterior approach can solve problems such as difficulty in rotation of the liver and bleeding control.

## Conclusions

Liver shape, size, and weight in a Chinese population vary over a considerable range. The distribution of small blood vessels on HIVC was varied. There is no consistent relationship between the diameter of the IRHV or SHV and the diameter of the RHV. The distribution of the hepatic vessels can be impacted by multiple factors, including liver function and anatomical variations. Therefore, it is difficult to infer the number and size of other vessels from the liver size or a single vessel. Because it is difficult to accurately and thoroughly determine the distribution of the HIVC by imaging studies such as ultrasound, angiography, CT scan, and MRI, it is impossible to accurately assess the number of small vessels prior to surgery. However, by studying liver specimens from a Chinese population, we found a pattern of HIVC distribution that can be used when creating the retrohepatic tunnel. In summary, the majority of the vessels drained into the middle and lower 1/3 of the left and front walls of the HIVC, with a slight predominance of drainage into the left wall. A few vessels drained into the upper 1/3 of the HIVC. Accordingly, it is easy to operate in the upper 1/3 of the HIVC; however, care must be taken when operating in the middle and lower 1/3 of the HIVC. Due to their lower location, the vessels should be managed using visualization that is as direct as possible. Based on the measured anatomical distances found in a Chinese population, a hemostat tip can almostly be passed through the space between the IRHV and the largest CV in front of the the HIVC until it reaches the space between the RHV and the MHV or their common trunk (vena cava fossa). Therefore, the use of the retrohepatic tunnel is a safe approach. Certainly, the blood vessels should be preserved as much as possible so as to preserve blood supply for the liver remnant and to protect liver function and provide compensatory function for subsequent possible diseases; thus, the right approach seems to be the best choice for passage of the hemostat tip through the retrohepatic tunnel.

## Materials and Methods

### Materials

A total of 32 formalin-fixed cadavers (including the diaphragm and the intact HIVC) were dissected in the Department of Anatomy of Southern Medical University. All of the harvested livers were intact adult (regardless of gender) livers in which no nodular sclerotic lesions or tumors were noted. Informed consent was obtained from the legal guardian of every cadaver. All methods were carried out in accordance with the provisions of the Declaration of Helsinki in 1995 (as revised in Edinburgh 2000). The study protocol was approved by Medical Ethics Committee of Southern Medical University.

### Instruments

The conventional surgical instruments used included hemostats, retractors, scalpels, scissors, and forceps. A custom-made 1-mm vessel probe was used. A caliper and a stainless steel ruler were used as measuring tools.

### Procedure

(1) The abdominal wall was incised to gain access to the abdominal cavity; the liver was observed grossly to determine whether significant lesions were present. The IVC was dissected above and below the liver. The upper IVC was divided at the level of the thoracic diaphragm, and the lower IVC was divided above the site of entry of the renal vein. (2) For specimen harvesting, the perihepatic ligaments were separated, the IVC was divided at the level of the diaphragm foramen and above the level of the renal vein, and the liver duodenal ligament was divided at the edge of the duodenum; in this way, the intact liver specimen was obtained. The surgeon carefully isolated the right, left, and middle hepatic veins at the upper edge of the liver in the IVC. The conditions of the sites of entry of the left, middle, and right hepatic veins into the IVC, as well as the HIVC, were observed. (3) A caliper was used to measure the relevant parameters of the liver and the HIVC. (4) The posterior wall of the HIVC was incised. Using three hypothetical (left, middle, and right) vertical lines, the wall of the HIVC was evenly divided into four great zones, i.e., the posterior (P), left (L), anterior (A), and right (R) zones; using two horizontal lines, the vessel wall was evenly divided into three great zones, i.e., the superior (S), medium (M), and inferior (I) zones. The vertical and horizontal lines crossed and evenly divided the vessel wall into 12 sub-zones. The right hepatic vein (RHV), the middle hepatic vein (MHV), the left hepatic vein (LHV), the caudate vein (CV), the SHV, and the inferior-right hepatic vein (IRHV) were exposed. The widths of the upper and lower openings and the length of the retrohepatic tunnel were measured, and the number and pattern of the distributed vessels were documented.

### Statistical analysis

The statistical software package SPSS 19.0 was used for statistical analysis. Quantitative data are expressed as mean ± standard deviation.

## Additional Information

**How to cite this article:** Zheng, W. *et al*. Anatomy of the retrohepatic tunnel in a Chinese population and its clinical application in liver surgery. *Sci. Rep.* **7**, 44977; doi: 10.1038/srep44977 (2017).

**Publisher's note:** Springer Nature remains neutral with regard to jurisdictional claims in published maps and institutional affiliations.

## Figures and Tables

**Table 1:** Various types of vascular branches accounted for their respective proportions (%).

Types of vascular branches	Single	Double	Triple	Quadraple
RHV	38.7	25.8	32.2	3.2
LHV	51.6	41.9	6.4	0
MHV	61.3	38.7	0	0
Posterior left superior edge vein	100	0	0	0
Posterior right superior edge vein	95.2	4.8	0	0
Right lateral posterior vein	91.7	8.3	0	0
Right dorsal vein	71.4	28.6	0	0
IRHV	70.4	29.6	0	0
CV	51.1	38.3	8.5	2.1
